# Long-Term Outcome of Potential Celiac Disease in Genetically at-Risk Children: The Prospective CELIPREV Cohort Study

**DOI:** 10.3390/jcm8020186

**Published:** 2019-02-05

**Authors:** Elena Lionetti, Stefania Castellaneta, Ruggiero Francavilla, Alfredo Pulvirenti, Giulia Naspi Catassi, Carlo Catassi, The SIGENP Working Group of Weaning and CD Risk

**Affiliations:** 1 Department of Pediatrics, Marche Polytechnic University, 60123 Ancona, Italy; giulia.catassi@gmail.com (G.N.C.); c.catassi@univpm.it (C.C.); 2 Department of Pediatrics, San Paolo Hospital, 70127 Bari, Italy; scastellaneta@libero.it; 3 Interdisciplinary Department of Medicine, Paediatric Section, University of Bari, 70126 Bari, Italy; ruggiero.francavilla@uniba.it; 4 Department of Clinical and Molecular Biomedicine, University of Catania, 95123 Catania, Italy; apulvirenti@dmi.unict.it; 5 Center for Celiac Research, Mass General Hospital for Children, Boston, MA 02114, USA

**Keywords:** celiac disease, potential celiac disease, natural history

## Abstract

Background: The long-term outcome of potential celiac disease (CD) is still a debated issue. We aimed to evaluate the progression of potential CD versus overt CD after 10-years of follow-up in a cohort of children genetically predisposed to CD. Methods: The CELIPREV study is prospectively following from birth 553 children with CD-predisposing HLA genes. Children with a diagnosis of potential CD continued to receive a normal diet and repeated the serological screening for CD every year. An intestinal biopsy was taken in presence of persistent positive serology. Results: Overall, 26 (4.7%) children received a diagnosis of potential CD (50% females, median age 24 months). All children were symptom-free. Twenty-three children continued a gluten-containing diet; at 10 years from the first biopsy, three children developed overt CD (13%), 19 (83%) became antibodies negative at 1 year from the first biopsy and remained negative up to 10 years of follow-up and one subject (4%) had fluctuating antibody course with transiently negative values and persistently negative biopsy. Conclusions: In children genetically predisposed to CD with a diagnosis of potential CD the risk of progression to overt CD while on a gluten-containing diet is very low in the long-term.

## 1. Introduction

Celiac disease (CD) is a systemic immune-mediated disorder caused by the ingestion of gluten-containing grains in genetically susceptible persons [1]. The phenotypic expression of the disease has changed over time with a marked decrease of classical cases and a striking increase of the atypical and subclinical phenotype [1]. Recent cohort studies in infants genetically at-risk of CD, periodically screened for CD autoimmunity, have shown a significant proportion of children affected with potential CD [2,3,4,5,6]. According to current definitions, potential CD is characterized by the presence of anti-transglutaminase (TGA2) and anti-endomysium (EMA) antibodies, with compatible HLA and without duodenal villous atrophy [7]. These patients may or may not have symptoms and signs of the disease and may or may not develop an overt CD with villous atrophy later on. Therefore, the need to start a gluten-free-diet (GFD), especially in asymptomatic patients, is still a debated issue [7]. The European Society of Paediatric Gastroenterology, Hepatology and Nutrition guidelines suggest that a seropositive, asymptomatic at-risk person, who does not show clear evidence of small intestinal mucosal damage, should be followed-up on a normal gluten-containing diet and re-evaluated at regular intervals [7]. Long-term prospective studies are needed to clarify the natural history of potential CD and its progression to overt CD. 

The Risk of Celiac Disease and Age at Gluten Introduction (CELIPREV) study is a multicentre, prospective study investigating the interplay between environmental and genetic factors on the development of CD in a cohort of infants with a family risk of CD, followed on from birth [2,3,8]. We have previously reported that in the CELIPREV study, the rate of progression of potential CD to overt CD by 2 years of follow-up was only 5% [3]. However, the long-term natural history of potential CD is still unclear. Here we report up to 10-years follow-up in CELIPREV children originally diagnosed as potential CD. 

## 2. Methods

The CELIPREV is a prospective, multicentre, nationwide intervention trial that was primarily aimed to evaluate the role of age at gluten introduction on CD development, in a large cohort of children at family risk of CD, that were followed from birth [2,3,8]. Family risk was defined by having at least one first-degree relative affected with CD. Newborns were recruited in 20 centres scattered throughout Italy between October 2003 and January 2009. Infants were assigned to introduce gluten-containing food at either 6 (group A) or 12 months of age (group B) by block randomization and then followed-up regularly. The study design and the characteristics of the CELIPREV cohort have been described previously [2,3].

For the purpose of the present study, we focused on the sub-group of children with a diagnosis of potential CD defined in the presence of: (a) a CD compatible HLA genotype (standard or high risk, see below); (b) a Marsh 0–1 lesion at the biopsy; and c) IgA TGA2 and EMA positivity or IgG TGA2 positivity in presence of IgA deficiency (IgA < 5 mg per decilitre). Children with potential CD were left on a normal diet unless symptomatic. Every 6 months for the first year and every year thereafter, antibodies and clinical conditions were checked and a small bowel biopsy was taken every 2 years in presence of persistent serological positivity or earlier if symptoms developed. A thorough dietary survey was performed by a dietician at each control visit to verify the intake of gluten.

The Institutional review board of each participating centre approved the study protocol, registered on ClinicalTrials.gov CELIPREV, number, NCT00639444. Written informed consent was obtained from the parents or guardians of the children.

### 2.1. HLA Genotype

The detection of HLA alleles was performed by the DQ-CD Typing Plus kit (BioDiagene, Palermo, Italy), as previously described [2]. On the basis of HLA determination, children were classified as having no risk of CD (the absence of HLA-DQ2 and HLADQ8), a standard risk of CD (a single or double copy of the DQB1*02 allele associated with DQA1 alleles different from the DQA1*05 or a single DQ2 (DQA1*05-DQB1*02) or DQ8 (DQA1*03-DQB1*0302/ 0305) haplotypes or a high risk of CD (homozygosity for DQA1*05-DQB1*02 or DQA1*05-DQB1*02-DQA1*0201-DQB1*02) [2,9].

### 2.2. Serologic Assay

Serum TGA2 was measured by means of an enzyme-linked immunosorbent assay with the use of a commercial kit (Menarini Diagnostics, Florence, Italy). More than 20 arbitrary units indicated a positive result. Results are expressed as multiple of the upper normal limit (UNL). EMA were detected by means of indirect immunofluorescence, with the use of monkey oesophagus as substrate (a titre of 1:10 or higher that resulted in a positive reaction was considered to be positive). Total serum IgA was measured by means of nephelometry.

### 2.3. Small-Bowel Biopsies 

Small-bowel biopsies were performed by means of upper endoscopy and at least 4 specimens were obtained from the bulb and the descending part of the duodenum. Lesions in the small intestine were graded at the coordinating centre in Ancona, Italy, according to the Marsh classification [10]. 

### 2.4. Outcome Measures

The primary outcome measure was the risk of overt CD at 10 years of follow-up after the diagnosis of potential CD. Secondary outcomes were: a) the natural history of CD-related antibodies in children with potential CD left on a normal diet; and b) to identify any possible discriminating factor between children with potential CD who developed overt CD from those who remained potential. 

### 2.5. Statistical Analyses

The dataset reflects follow-up of the cohort as of June, 2018. Data are expressed as mean ± SD. Proportions were compared with the use of the χ^2^ test with Yate correction for continuity or Fisher exact test as appropriate; comparison of continuous variables was performed with the use of Student *t* test. Survival analysis was performed to observe the occurrence of the event (overt CD) over long periods of time. Kaplan–Meier curves were plotted for the primary end point (i.e., the risk of overt CD according to age). All differences were considered to be statistically significant at a 5% probability level and all reported P values were 2- sided. Statistical analysis was performed with the use of tools for survival analysis and recursive partitioning analysis within the R system.

## 3. Results

### 3.1. Patients

**Figure 1:**
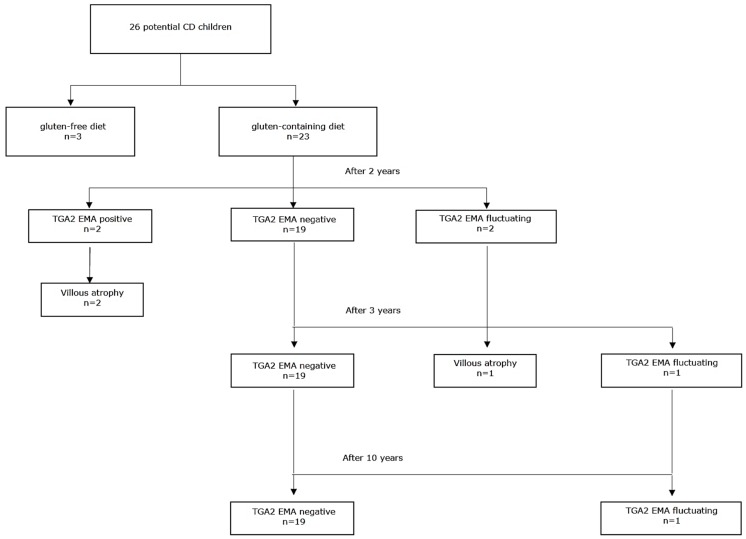
Flow diagram of potential celiac disease (CD) children.

After exclusion of 125 patients who dropped out, the cohort included 707 infants. Of them, 154 were negative for HLA-DQ2 and HLA-DQ8 and were excluded from further follow-up. The final study group included 553 children who were positive for HLA-DQ2, HLA-DQ8 or both. Among them, 26 (4.7%) children received a diagnosis of potential CD. They were 13 (50%) females, with a median age of 12 years (range, 11.3 to 13.7) in June 2018, when the follow-up was stopped. The median age at diagnosis of potential CD was 24 months (range, 15 to 60). By the end of the study, all children had at least 10 years of follow-up after the diagnosis of potential CD. Of these 26 children, all were IgA TGA2 and EMA positive, except one with IgA deficiency who was positive for TGA2 of IgG class. Fifteen children had a Marsh score 0 (58%) and 11 (42%) had a Marsh score 1. HLA was at high risk of CD in 7 (27%) children and at standard risk in the remaining 19 (73%). The mean TGA2 value was 5.2 UNL. All children were symptom-free, had normal nutritional parameters and none had other autoimmune disorder. Figure 1 shows the flow diagram of the 26 children with potential CD. Three patients started a GFD because of parents’ choice. Twenty-three children continued a gluten-containing diet. During follow-up, the amount of daily gluten intake was normal (at least 15 g per day) in all cases. We obtained serological data at 10 years of follow-up of all 23 children on gluten-containing diet.

### 3.2. Study Outcomes

**Figure 2:**
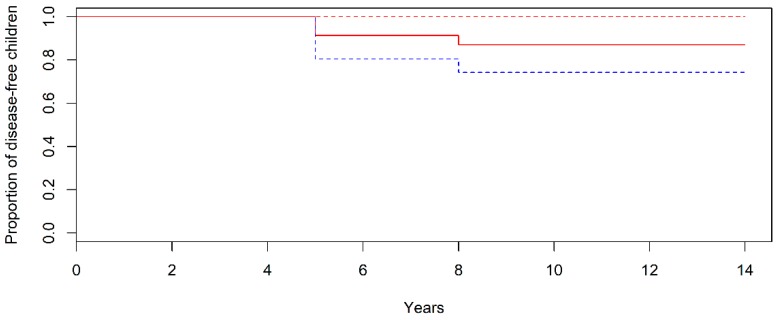
Kaplan–Meier Estimates of Overt Celiac Disease in Children with Potential Celiac Disease According to Age. Dashed lines represent 95% confidence intervals.

Overall, 10 years after the first biopsy, only three out of 23 children with potential CD on gluten-containing diet developed overt CD (13%). Figure 2 shows the proportion of children developing overt CD, according to age. Of the three patients that developed overt CD, two had persistent TGA2 and EMA positive at each time of follow-up and showed villous atrophy two years after the first biopsy at age 5 years; the third child had fluctuating EMA and TGA2 titre with transiently negative values during follow-up and showed villous atrophy 3 years after the first biopsy at the age of 8 years. 

Out of the other 20 children, 19 (95%) became TGA2 and EMA negative at 1 year from the first biopsy and remained negative up to 10 years of follow-up. One subject (5%) had fluctuating TGA2 course with transiently negative values. In this case the intestinal biopsies performed after 2 and 4 years of follow-up still showed a Marsh score 0. All 20 children were still symptom-free and did not developed any autoimmune disease at the 10 years follow-up.

Of note, out of the three children that started a gluten-free diet because of parents’ choice, one developed an autoimmune thyroiditis.

The demographical, clinical, serological and genetic characteristics of this study-group are shown in Table 1. There was no significant difference between children developing CD and the others for all the investigated variables. Only the serological outcome had a discriminating power; indeed, the persistent positivity was present in 2 children both developing overt CD as compared to none of the other children (*p* < 0.001); a serological remission was observed in 19 out 20 (95%) that did not develop CD compared to none of those developing overt CD (*p* < 0.001).

## 4. Discussion

The present study shows that in children genetically at-risk for CD with a diagnosis of potential CD the risk of progression to overt CD while on a gluten-containing diet is very low in the long-term. 

The results from this longitudinal study provide unique data on the natural history of potential CD evaluated from the onset of the disease and have several implications for clinical practice. Potential CD is, indeed, an emerging condition, with a still unclear pathogenesis in comparison to overt CD. According to the recent ESPGHAN guidelines, children with a CD-affected first-degree relative should be screened by HLA testing and TGA2 test [7]. We previously showed that in this group of children the prevalence of potential CD is high [2,3] and its management is still controversial: (a) the observation that CD autoimmunity may spontaneously normalize in children with potential CD while on a gluten-containing diet would suggest caution before starting a GFD for life without a conclusive proof of a gluten-induced disease [3,11]; (b) potential CD may be the first step of the disease, that would be overly manifested later in time and starting a GFD as soon as possible may prevent the risk of an underling disease in the long run [12]. Very few studies have analysed long term risks related to a gluten containing diet in these patients. 

In 2010, Kurppa et al. [12] found that 7 out of 8 children with potential CD developed overt CD after 2 years of a gluten containing diet. In the Finnish study all children were symptomatic, aged between 4 and 17 years, referred from primary health care because of suspicion of celiac disease [12]. In 2014, Auricchio et al. [11] reported the 9-year follow-up of a large cohort of children with potential CD showing that 32% of those on a gluten-containing diet progressed to villous atrophy. The study cohort included either genetically at-risk children diagnosed by screening early in life or older children tested for CD for other reasons. A recent study evaluating the effect of GFD in a cohort of 65 symptomatic children with potential CD showed that symptoms after GFD improve in only about half of the cases [13]. Therefore, the authors suggested caution before starting a GFD in this condition. 

In adults, Biagi et al. [14] retrospectively evaluated 47 patients with potential CD. Of them, 24 patients followed a gluten-containing diet and 5 (24%) developed a flat mucosa, while the others maintained a normal duodenal mucosa for many years and their symptoms spontaneously improved [14]. Volta et al. [15] described the natural history of 77 adults diagnosed with potential celiac disease; gluten withdrawal led to significant clinical improvement in all 61 symptomatic patients. The 16 asymptomatic patients continued on gluten-containing diet and only 1 (6%) developed mucosal flattening [15].

We firstly described the natural history of potential CD in young asymptomatic children screened for family risk of CD, showing that only 5% of them developed overt CD after 2 years of follow-up [3]. A prolonged follow-up was, however, required to exclude that further changes in gluten tolerance might take place over time in these patients. Now we show that in the same well-defined cohort of children with potential CD, only 13% developed villous atrophy after 10 years of follow-up, therefore confirming that the finding of CD autoimmunity only rarely predicts progression to overt CD. Worth noting, children with potential CD left on a gluten-containing diet did not develop any other autoimmune disease during the 10-year follow-up, while an autoimmune thyroiditis developed in one of the few patients put on a GFD due to parental choice, a finding that does not support the hypothesis that a GFD is a safer choice than a regular diet in children with potential CD. 

Our finding of a lower percentage of progression of potential to overt CD as compared to previous studies may be explained by: (a) the age of our study-group; indeed, this is the first study including only young children, who may have a transient antibodies positivity in the first years of life more frequently than older children; (b) the study design; in our cohort study per protocol children at family risk were periodically screened for CD autoimmunity, thus allowing to detect transient antibody positivities that would not be seen in real life. 

With the aim to identify any possible indicator of progression from potential to overt CD, we found that the persistence positivity of TGA2 and EMA during follow-up was the only factor that significantly predicted the development of overt CD in our cohort. On the other hand, the spontaneous loss of serum CD autoimmunity (TGA2 and EMA) was the most frequent outcome in children who did not develop CD, while the possibility of fluctuating antibodies was observed in both groups. These results are consistent with several previous studies showing transient CD autoimmunity in children with serial testing [3,6,11,16,17,18,19,20]. Our study definitively shows that in these subjects with spontaneous loss of TGA2 and EMA, CD autoimmunity truly abates and tolerance persists over time. On the contrary, the presence of persistent positivity needs more attention because the risk of developing overt CD is very high in these cases. 

The finding of CD related autoantibodies in patients bearing the HLA predisposing genes without mucosal damage suggests that these children developed an adaptive anti-gluten immune response but they possibly lacked the innate activation of epithelial cells eventually leading to villous atrophy [21]. It is possible to speculate that in children with transient TGA2, Tregs cells have gained the upper hand [22]. A lower level of pro-inflammatory adaptive anti-gluten immunity might also be responsible for lack of villous atrophy [21]. 

We know that a prolonged follow-up of this cohort is still needed and further studies are required to better explore which factors may influence the balance between tolerance and immune-response to gluten finally leading to the progression to overt CD. 

## 5. Conclusions

Our study has three important implications: (1) in children with a family risk of CD there may be a state of temporary positivity of celiac serology, thus it is always mandatory to back up a positive serology with a small-intestine biopsy; (2) in the absence of the histological confirmation of the disease, a gluten containing diet can be safely recommended, with a clear indication to attend a careful serological follow-up; (3) in presence of persistent serological positivity a further biopsy is strongly required; in case of spontaneous serological remission, overt CD is unlike to develop. 

The demonstration of a self-limiting flare-up of CD serology raises an intriguing question: how does this spontaneous remission occur? If nature can, can we? Future research will now design experiments addressing this question. 

## Figures and Tables

**Table 1:** Demographical, clinical, serological and genetic characteristics of children that developed overt celiac disease and children that remained potential.

	Not-Developing Overt CD (*n* = 20)	Developing Overt CD (*n* = 3)
**Median age at diagnosis, mo**	24	36
**Female sex, no. (%)**	10 (50)	2 (66.7)
**First-degree relative with CD, no. (%)**		
**Type of relative**		
Father	1 (5)	2 (66.7)
Mother	9 (45)	0
Brother	2 (10)	0
Sister	8 (40)	1 (33.3)
**Breastfed, no. (%)**	16 (80)	1 (33.3)
**Duration of breastfeeding, mo**	6.3 ± 5.8	9 ± 15.6
**Introduction of gluten, no. (%)**		
At 6 mo, Group A	13 (65)	1 (33.3)
At 12 mo, Group B	7 (35)	2 (66.7)
**TGA2 level at diagnosis, xUNL**	5.0 ± 4.6	4.7 ± 4.3
**Caesarean delivery, no. (%)**	7 (35)	2 (66.7)
**HLA genotype, no. (%)**		
Standard risk	14 (70)	1 (33.3)
High risk	6 (30)	2 (66.7)
**Marsh score at first biopsy, no. (%)**		
Marsh 0	13 (65)	1 (33.3)
Marsh 1	7 (35)	2 (66.7)
**Serology outcome (TGA2 and EMA), no. (%)**		
Persistent positivity *	0	2 (66.7)
Fluctuation	1 (5%)	1 (33.3)
Spontaneous remission *	19 (95%)	0

Plus-minus values are expressed as means ±standard deviation. UNL denotes upper normal limit. CD: celiac disease; TGA2: anti-transglutaminase antibodies; EMA: endomysium antibodies. * *p* < 0.001 for the comparison of both persistent positivity and spontaneous remission between the groups.
